# Digital mapping of a manual fabrication method for paediatric ankle–foot orthoses

**DOI:** 10.1038/s41598-021-98786-z

**Published:** 2021-09-24

**Authors:** Joyce Zhanzi Wang, Jonathon Lillia, Muhannad Farhan, Lei Bi, Jinman Kim, Joshua Burns, Tegan L. Cheng

**Affiliations:** 1 University of Sydney School of Health Sciences, Faculty of Medicine and Health & Children’s Hospital at Westmead, University of Sydney, Sydney, NSW Australia; 2 EPIC Lab, Kids Research, The Children’s Hospital at Westmead, Westmead, NSW 2145 Australia; 3 Faculty of Medical Rehabilitation Science, Taibah University, Al Madinah Al Munawarah, Saudi Arabia; 4 School of Computer Science, Faculty of Engineering, University of Sydney, Sydney, NSW Australia

**Keywords:** Health care, Medical research, Computational biology and bioinformatics, Musculoskeletal system

## Abstract

Ankle–foot orthoses (AFOs) are devices prescribed to improve mobility in people with neuromuscular disorders. Traditionally, AFOs are manually fabricated by an orthotist based on a plaster impression of the lower leg which is modified to correct for impairments. This study aimed to digitally analyse this manual modification process, an important first step in understanding the craftsmanship of AFO fabrication to inform the digital workflows (i.e. 3D scanning and 3D printing), as viable alternatives for AFO fabrication. Pre- and post-modified lower limb plaster casts of 50 children aged 1–18 years from a single orthotist were 3D scanned and registered. The Euclidean distance between the pre- and post-modified plaster casts was calculated, and relationships with participant characteristics (age, height, AFO type, and diagnosis) were analysed. Modification maps demonstrated that participant-specific modifications were combined with universally applied modifications on the cast's anterior and plantar surfaces. Positive differences (additions) ranged 2.12–3.81 mm, negative differences (subtractions) ranged 0.76–3.60 mm, with mean differences ranging from 1.37 to 3.12 mm. Height had a medium effect on plaster additions (r_s_ = 0.35). We quantified the manual plaster modification process and demonstrated a reliable method to map and compare pre- and post-modified casts used to fabricate children's AFOs.

## Introduction

**Figure 1:**
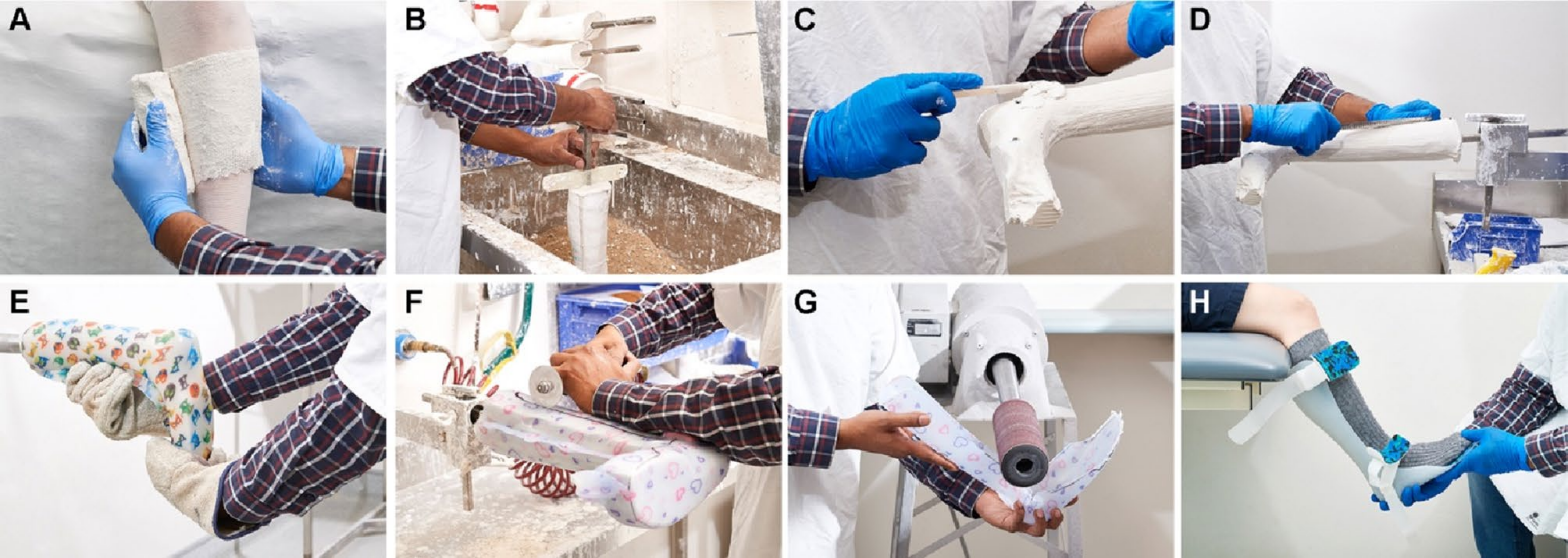
Traditional AFO fabrication involving plaster moulding. (**A**) Creating the negative cast of a person, (**B**) filling the negative cast with liquid plaster to produce the positive cast, (**C**) applying additional plaster modifications, (**D**) refining plaster modifications, (**E**) vacuum forming polypropylene over modified cast, (**F**) cutting the polypropylene AFO from the cast, (**G**) finishing the AFO and (**H**) fitting the AFO to a person.

Ankle–foot orthoses (AFOs) are commonly prescribed external lower limb orthoses that encompass the foot, ankle and lower leg^1,2^. AFOs are prescribed to manage walking difficulties in people with neuromuscular, musculoskeletal and cerebrovascular conditions^3^. There are five common types of AFOs: fixed AFO, hinged AFO, posterior leaf spring-AFO (PLS-AFO), ground reaction AFOs, and supra malleolar orthosis (SMO). The traditional manufacturing of custom AFOs (Fig. 1) involves a plaster moulding technique that requires highly skilled orthotists and technicians, dedicated plaster facilities and large volumes of consumables. Fabrication involves plaster modifications (Fig. 1C,D) introduced based on the mechanical and deformity corrections required by a patient. The labour-intensive fabrication processes can lead to long waiting periods and are limited in design options. Excessive waiting periods for children can lead to them outgrowing their devices rapidly, and limited design choices has been found to lead to user dissatisfaction and negative feelings related to use and appearance^4–6^. To address the shortfalls in traditional production processes, digital workflows that include 3D scanning and 3D printing have emerged as viable alternatives for AFO fabrication^7–9^.

A digital workflow is a feasible method of production AFOs, with a recent systematic review finding that custom 3D printed AFOs were comparable to traditional AFOs in terms of biomechanical outcomes, including temporal-spatial gait parameters^9^. Earlier studies have shown the potential for computer-aided design and manufacturing (CAD/CAM) techniques for fabricating orthotic devices to be more efficient than traditional plaster moulding. However, the learning curve for orthotists using a CAD/CAM system can take up to 4 years^10^, with working time mostly taken up by the design and modification phase^11^. With a better description and translation of plaster modification into the digital realm, there are efficiencies to be gained in AFO fabrication. For instance, a randomised clinical trial comparing a CAD/CAM process with traditional manufacture for children's AFOs did not support the digital process with respect to saving time or device quality^12^. The study found that computer-based modification and design led to higher proportion of devices failing to meet specification, with 17% of CAD/CAM devices reported as having fit-based issues requiring remaking of the cast (vs. 3% of traditional AFOs). The authors highlighted that their digital processes show promise but that required orthotist training on making computer-based modifications. From this work, it appears that modifications are the weakest link in the AFO digital fabrication process, therefore more work is needed to describe and translate plaster-based modification processes.

**Figure 2:**
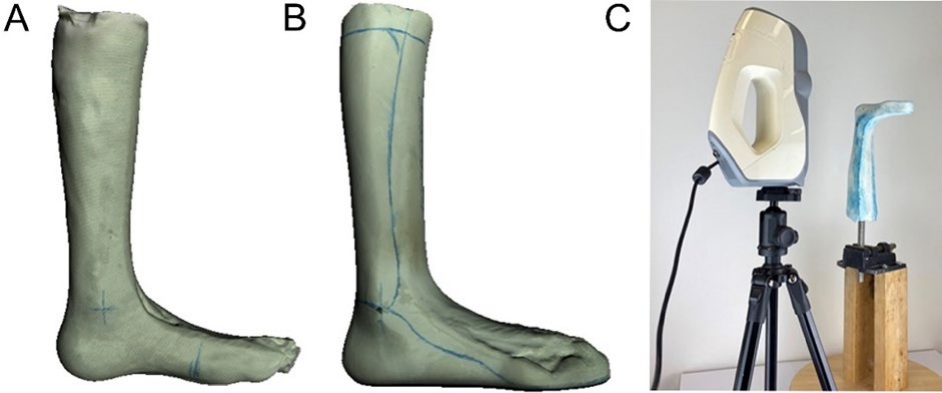
(**A**) Pre-modified plaster cast, (**B**) Post-modified plaster cast with trimlines drawn in blue and (**C**) the set up used to 3D scan casts.

Manual cast modifications had been imitated digitally, ranging from crude methods of expanding the scanned limb by a set value to proprietary methods that integrate skeletal structures. However, existing methods may not consider the constraints of complex anatomy and severe disease states. For example, a workflow proposed by Robert et al. universally added 3 mm to the malleoli, with other modifications included on request, resulting in problems with over-fitting of CAM AFOs, especially around the ankle^12^. Moreover, prior studies on digital cast modifications were combined with trimline generation (how much of the lower leg the AFO covers, which determines the stiffness of the device; shown in Fig. 2B) and building 3D geometry when generating AFOs^13^. However, to our knowledge, no studies have digitally translated the manual modification process involved with AFO fabrication.

We suggest that mapping an orthotist's actions during traditional manufacturing could be an important method of visualising and documenting the modification process, with implications in clinician education and evidence-based translation to CAD modification. Thus, this study aims to take the first steps in mapping and digitising an orthotist’s action during plaster cast modification for the fabrication of children's AFOs. The general location and magnitude of plaster modifications as well as their relationship with participant characteristics (age, height, AFO type, and diagnosis) were explored.

## Materials and methods

### Participants and research design

Plaster casts of children's (N = 50) lower limbs were obtained from the Orthotics Department in the Children’s Hospital at Westmead, Sydney, Australia, in accordance with an approved human ethics protocol (Sydney Children’s Hospitals Network Human Research Ethics Committee, protocol LNR/17/SCHN/242). The study was performed in accordance with the Declaration of Helsinki and informed written consent was gained by the participant and/or the legal guardian of the participant. Any child prescribed AFO at the hospital was eligible for inclusion and in children with a bilateral prescription, one side was randomly chosen. The height (N = 42), age (N = 50) and condition the child was prescribed an AFO (N = 48) were recorded.

To take an impression, the orthotist wrapped plaster bandages around the lower leg in a non-weight-bearing position. In some cases, manual deformity correction was applied as the plaster bandages dried with any further corrections required applied before 3D scanning. The bandages were removed (Fig. 1A) and filled with plaster to form a positive cast—this was defined as the 'pre-modified cast' (Fig. 1B). The plaster-modified cast used for thermo-vacuum forming of the traditional AFO was considered the 'post-modified cast' (Fig. 1D). Modifications made by the experienced orthotist (5 years) were assumed to be correct.

### Data collection

The pre- and post- plaster casts were scanned using a handheld white structured light 3D scanner (Artec Eva, Artec Group, Luxembourg). Artec Studio software package (Version 11; Artec Group, Luxembourg) was used to generate 3D models. Both pre-modified and post-modified casts (Fig. 2A,B) were scanned at 16 frames per second within a working distance between 0.4 and 0.6 m. The scanner was placed on a tripod with the cast placed on a rotating stand (Fig. 2C). For most cases, more than two scans were required to capture the entire cast surface, which were aligned and meshed during post-processing.

### Mesh registration and comparison

Mesh registration was conducted using 3-Matic (Mimics Innovation Suite version 12.0, Materialise, Leuven, Belgium) to align the pre- and post-modified 3D models via an iterative closest point algorithm. The registration was performed based on marks made on the plaster casts by the orthotist.

The paired registered casts were compared in opensource CloudCompare software (2.9.1) by bidirectional Euclidean surface to surface distance^14^. The 'Mesh to Mesh Difference' (MMD) is defined as the value of the Euclidean surface-to-surface distance between a 3D shape (pre-modified cast) and a reference 3D shape (post-modified cast), with positive numbers indicating plaster addition and negative numbers indicating plaster subtractions to the pre-modified cast. These values are represented numerically by the mean value of MMD (mMMD), the average value of positive values of MMD (pMMD) and the average value of negative values of MMD (nMMD) for each pair. The MMD can also be visualised with a “modification map” that illustrates the direction and magnitude of change between the 3D shapes using a two-colour scheme projected on the pre-modification cast.

### Intra-rater reliability

**Figure 3:**
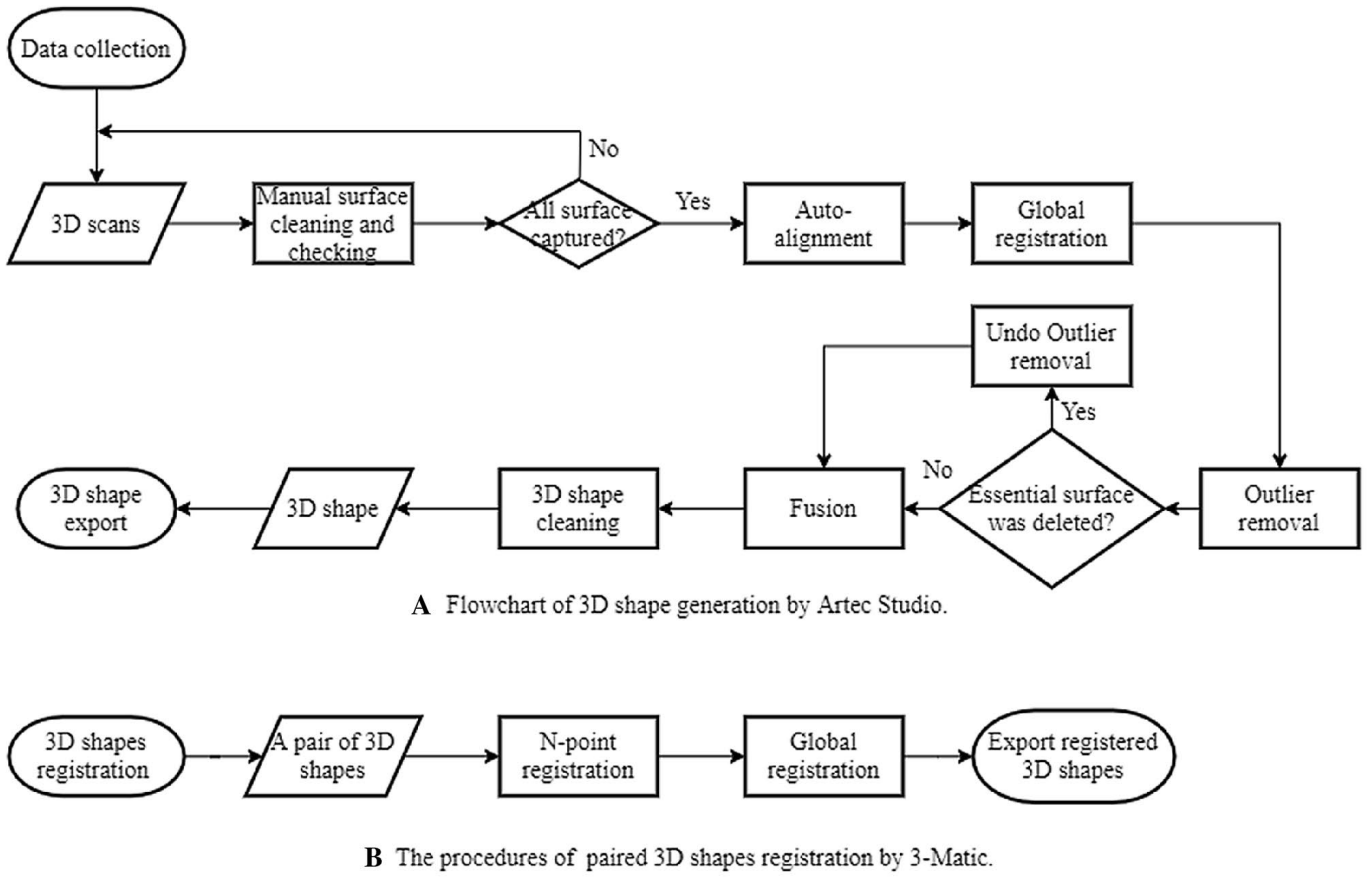
Flowcharts. (**A**) 3D shape generation by Artec Studio. (**B)** 3D shape registration by 3-matic.

Two intra-rater reliability studies were conducted to ensure that 3D scanning of the casts and the post-processing and registration workflows were reproducible. Ten casts (five pre-modified and five post-modified casts) were scanned twice using the same protocol (Fig. 3A) on different days. The volume (mm^3^) and surface area (mm^2^) of each 3D shape were used to assess the reliability of the scanning process. Following this, ten unregistered pairs of pre- and post-modified casts were selected and registered in 3-Matic twice using the same protocol (Fig. 3B), an hour apart. Registered pairs were compared in CloudCompare with the outcome values of maximum pMMD, nMMD and mMMD used to assess the reliability of the registration process.

### Statistical analysis

Intraclass correlation coefficient (ICC) was used to evaluate reliability. The two-way mixed-effects model with a 'single rater' type and an absolute agreement was used in this study. ICC values closer to 1.00 indicate more robust reliability, with values greater than 0.90 considered excellent^15^. Further statistical analysis was conducted using GraphPad Prism 7 for Windows 10 (GraphPad Software, San Diego, USA). Non-parametric tests (Kruskal–Wallis ANOVA) were chosen to compare the means in groups of pathology and AFO type^16^, with α was set at 0*.*05. A Spearman correlation coefficient, *r*_*s*_, was used to determine the strength and direction of the relationship between variables^15^. The *r*_*s*_ with a range of 0.00–0.25 represents little or no relationship, 0.25–0.50 determines the fair relationship, 0.50–0.75 represents moderate relationship, and the excellent relationship is indicated by the value above 0.75^15^. In addition, the effect size can also be represented by the correlation coefficient. Portney^15^ has also denoted that small effects can be seen with r_s_ greater than 0.10 but smaller than 0.30, *r*_*s*_ ranged from 0.30 to 0.50 indicates medium effects and the large effects can be shown when *r*_*s*_ is greater than 0.50.

## Results

### Participants

Fifty participants aged 1–18 years old (31 males, 19 females) were recruited. The average age was 8.6 years, with a standard deviation (SD) of 3.5 years. Participants were divided into five groups based on pathology: cerebral palsy (CP, N = 25), congenital talipes equinovarus (CTEV, N = 6), spina bifida (SB, N = 5), neurofibromatosis type 1 (NF1, N = 2) and 'Others' (N = 12). Participants diagnosed with an idiopathic condition or where a pathology only had one participant registered were classified as ‘Others’. Two participants who didn't have their pathology recorded were also classified as ‘Others’. There were four types of AFOs prescribed for this cohort, including fixed AFO (N = 30), hinged AFO (N = 16), PLS-AFO (N = 2) and SMO (N = 1). The AFO type of one participant was not recorded.

### Reliability of 3D scanning and registration

The intra-rater reliability of two phases of the workflow were assessed: 3D scanning and mesh registration. For 3D scanning, the ICC_(3, 1)_ of volume and area measures were calculated as 0.9996 and 0.9994 respectively. The average percentage errors of volume and surface area are 0.44% (SD 0.36) and 0.34% (SD 0.16), demonstrating a strong reliability of our cast 3D scanning process. For registration, the ICC_(3, 1)_ of maximum nMMD, maximum pMMD and mMMD were 0.99998, 0.99999 and 0.99999 respectively. The average percentage errors of maximum nMMD (0.05%, SD 0.13) and maximum pMMD (0.07%, SD 0.19), and mMMD (0.06%, SD 0.15) have also demonstrated the strong reliability of manual mesh registration using 3-Matic.

### Modification map

**Figure 4:**
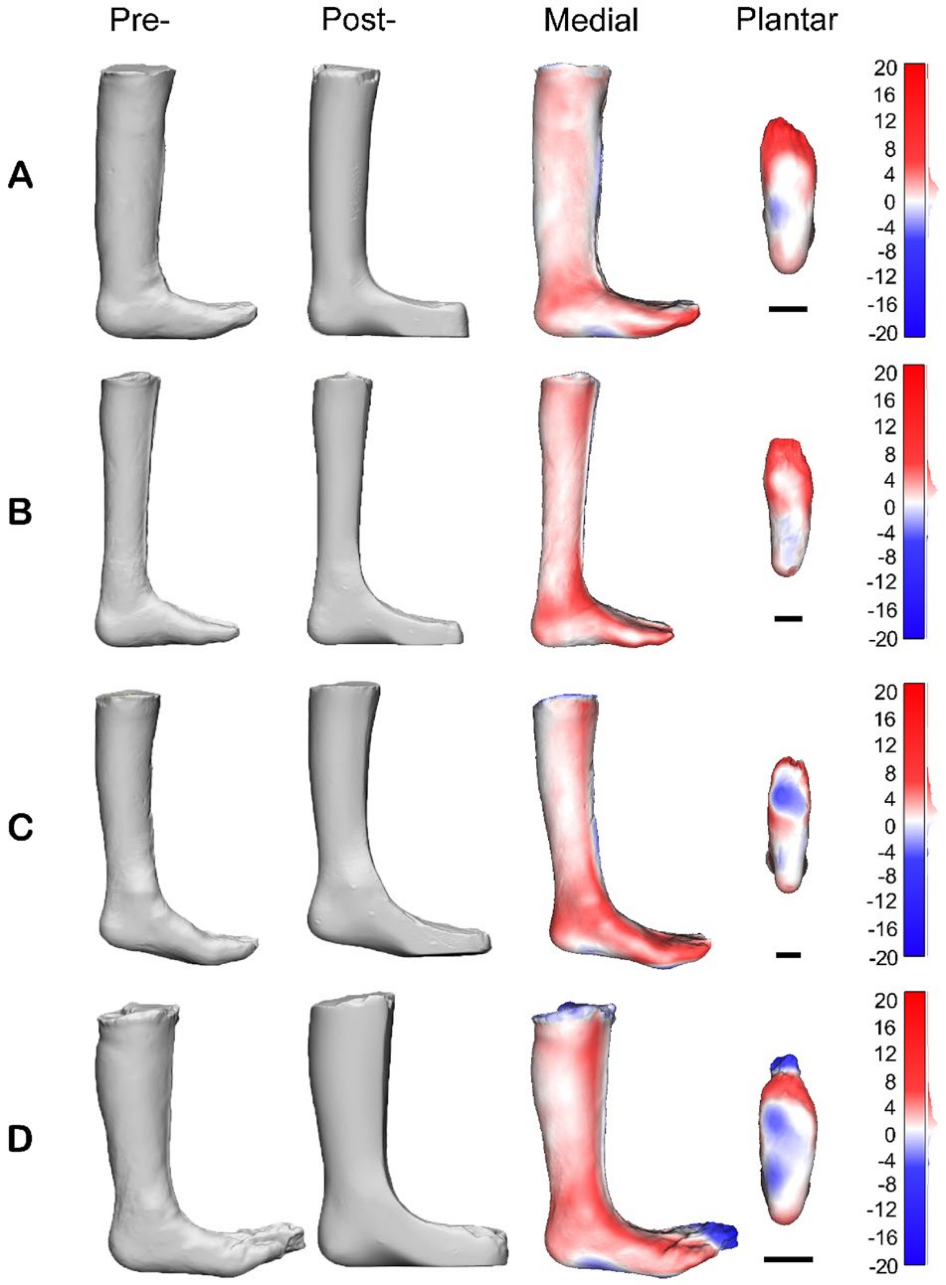
The medial view of the pre- and post-modification casts, and a medial and plantar view of the modification maps of four representative participants who were prescribed AFOs (unit: mm). Participant (**A**) was classified as NF1 and received a fixed AFO, participant (**B**) was classified as CP and received a fixed AFO, participant (**C**) was classified as other and received a fixed AFO, and participant (**D**) was classified as CP and received a hinged AFO. The modification maps represent the difference between the pre- and post-modification casts, with red colours (positive values) representing plaster additions and blue colours (negative values) representing plaster subtractions from the pre-modification cast. Scale bar represents 50 mm.

The modification map represents the 3D pattern of plaster changes between the pre- and post- modified casts projected onto the pre-modified cast. The modification maps of four examples can be seen in Fig. 4, with red shades representing areas where plaster was added to the pre-modified cast and blue shades representing areas where plaster was removed.

Regions of plaster addition were most commonly found around the toes, and the medial and lateral borders of the anterior of the cast that were squared off. Moderate amounts of plaster were added around the area of ankle and heel, however, little plaster added to the bony prominences of either the medial or lateral malleoli. Small amounts of plaster were added around the calf to smooth wrinkle artefacts, which were more evident in smaller sized casts. No distinct additions were applied to the navicular, heads of the first and fifth metatarsals, or the base of the fifth metatarsal. Plaster subtractions were consistently applied to the foot plate to flatten the plantar surface, round the “toe box” and to the dorsal surface of the foot to remove casting artefacts. Some casts that were in plantar flexion had plaster subtracted from the plantar surface of the forefoot to correct the ankle into a 90-degree position (Fig. 4D).

### Mesh-to-mesh difference (MMD)

**Figure 5:**
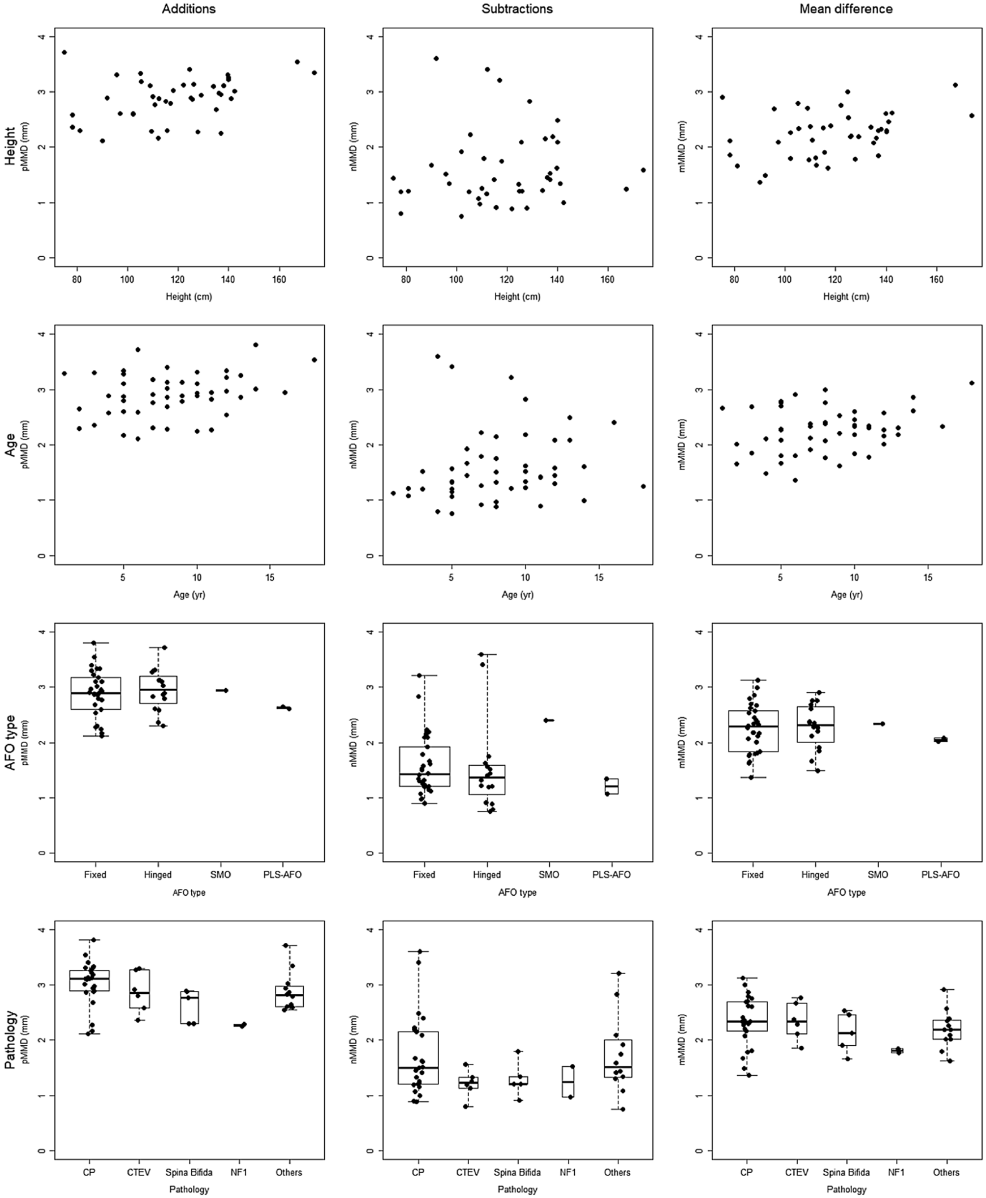
Comparing participant characteristics (rows) with plaster additions, subtractions and mean modifications between pre- and post-modified casts (columns). Rows describe participant characteristics, such as age, height, AFO type and pathology. Columns represent mesh-to-mesh distance (MMD), as average value of positive MMDs (pMMD), average value of negative MMDs (nMMD) and the mean value of MMD (mMMD) of each cast. SMO, supra malleolar orthosis; PLS-AFO, posterior leaf spring ankle–foot orthosis; CP, cerebral palsy; CTEV, congenital talipes equinovarus; NF1, neurofibromatosis type 1.

The pMMD, nMMD and mMMD of each pair were compared with participant height, age, pathology and prescribed AFO types (Fig. 5). Across our cohort, the pMMD was 2.90 mm (range 2.12–3.81 mm), the nMMD was 1.59 mm (range 0.76–3.60 mm), and the mMMD was 2.26 mm (range 1.37–3.12 mm). The AFO casts with the maximum and minimum pMMD and mMMD values were made for participants diagnosed with CP who were prescribed fixed AFOs. However, maximum nMMDs was found in a participant classified as CP and the minimum nMMD was found in a participant classified as 'Other', with both prescribed hinged AFOs.

Fair direct correlations have been revealed between participant’s height and pMMD (r_s_ = 0.35, *p* < 0.05) and mMMD (r_s_ = 0.31, *p* < 0.05), whereas nMMD was not linked to height (r_s_ = 0.18, *p* = 0.26). No relationships have been found between participant’s age and pMMD (r_s_ = 0.26, *p* = 0.07), nMMD (r_s_ = 0.26, *p* = 0.06) and mMMD (r_s_ = 0.21, *p* = 0.14).

The pMMD, nMMD, and mMMD were comparable across all prescribed AFO types. Analysis with one-way ANOVA found no significant differences between the four AFO type groups when looking at pMMD (*p* = 0.65), nMMD (*p* = 0.34) or mMMD (*p* = 0.75).

Between the pathology groups, analysis with one-way ANOVA found no significant difference in pMMD (*p* = 0.12), nMMD (*p* = 0.28) or mMMD (*p* = 0.28).

## Discussion

This study demonstrated a reliable method that characterizes and creates a digital map of plaster cast modifications during traditional AFO manufacturing. We were able to visualise an orthotist's actions during the fabrication of children's AFOs by comparing the 3D scans of casts taken pre- and post-modification. In comparing participant characteristics with the amounts of plaster added and subtracted, a medium effect was found between the height and both plaster additions and mean plaster modification. We suggest that our results are the first step in decoding the craftsmanship of orthotist modifications. These results could be used to inform teaching practices, for clinicians to visualise and monitor their own modifications and create reference maps for digital fabrication.

Our study found that the orthotist's plaster modifications were generally a process of smoothing, with more plaster added than removed. This is more nuanced than previously published approaches of enlarging 3D scans by a set value when making digital AFO models^12^. In line with anecdotal experience of orthotists, we found that there were key modifications made to all casts, although there is an absence of literature in this area. The universal modifications include squaring the anterior surface, adding plaster to the malleoli, extending and rounding the “toe box” to facilitate polypropylene thermo-vacuum forming, AFO demoulding, and to make the final device easy to don and doff. When looking at MMD values, we found participant's height but not age, has medium effects on orthotist's modifications. This observation aligns with the findings that the participant's height is directly correlated to the knee height rather than their age, especially for participants with CP^17^. There were no distinct differences in additions, subtractions or average plaster modifications between the five pathology classifications. This was surprising, as we anticipated different therapeutic goals, such as the correction of plantarflexion, inversion or internal forefoot rotation, would be reflected in the MMD values. However, the MMD values may not be sensitive enough to represent spatial differences between plaster modifications. The wide range of plaster additions and subtractions in the CP group could be due to the heterogeneity of the condition, which includes four main types of foot deformities^18^, and may warrant further analysis. Further, the large variations in plaster modification within the 'other' pathology group could be due to the complexity of these lower limb deformities and requirements. Our modification map suggests that plaster cast modifications are a patient-specific process for customised AFO fabrication, which could be potentially catalogued by participants' original foot deformities or conditions.

Our study is the first in reporting a digital modification map applied in the field of orthotics. However, digital modification maps have been used to show differences between 3D shapes in other fields of medicine and health, including prosthetics, studies on anatomy and medical devices aesthetics. Steer et al. established a software to compare 3D surface scans of residual limbs, analysing shape variation across population and quantifying changes amongst the prosthetic sockets^19^. Stanković et al*.* were able to use heatmaps overlayed onto 3D scans of participant's feet to identify foot deformities^20^. In orthopaedics, heatmaps were used to visualise the differences between the shape of a bone defect and the normal anatomy, such as in scapulae, femorae and tibiae^21,22^. Lu et al*.* used heatmaps applied to 3D scans of people to predict body composition and whole-body fat percentages^23^. Muggli et al*.* used heatmaps to model the association between prenatal alcohol exposure and craniofacial shape in foetal alcohol spectrum disorder^24^. Digital heatmaps have also been used to predict the changes in facial soft tissues from wearing dentures^25,26^. However, no studies have utilised the heatmap to identify the impact of a clinician's action on the fabrication of orthotic devices, specifically on a cast of a person.

There are several limitations to our study and directions for future research. A key limitation is the sample size of our database (N = 50), with small numbers in subgroups, such as PLS-AFO (N = 2) and NF1 (N = 2). Further, only one orthotist was involved, limiting our ability to draw conclusions for all plaster cast modifications, as this process can be stylistic and may vary between orthotists. As a specialised orthotist in a children's hospital, we assumed that our orthotist made modifications correctly and that the final AFO was appropriate for the participant. Further studies will be required to expand the database with more participants and include more orthotists. We also did not define regions of interest on the casts as other studies have done for spinal orthoses. Wong et al*.* compared conventional and CAD/CAM methods of modifications for spinal orthoses amongst five interested regions, namely axilla, thoracic, lumbar, abdominal and pelvic regions^11^. In the future, an approach is required to define and separate regions of interests of casts consistently for visualising more specific modifications. We found that participant height had the strongest relationship to cast additions, however due to our limited data collection, we could not explore the relationships with other variables, such as foot length, degree of foot deformity, or foot posture index. Indeed, the relationships between cast modifications and the clinical variables in this study may be more distinct with a larger database, with factors such as foot length and deformity revealing more insights. In addition, we made the assumption that the unmodified plaster cast was a true representation of the participant’s lower limb—future studies will include direct scans of the lower leg.

## Conclusion

This study mapped and digitised the behaviour of an orthotist during the process of traditional cast modification for children's AFOs. The modification map has successfully visualised and quantified the locations of the orthotist's plaster cast modifications, demonstrating the principles behind traditional production processes graphically. Cast modifications were both regular and participant specific, with plaster added and subtracted to smooth the surface and build a regular shape. From the variables assessed in this study, participants' height had a medium effect on plaster additions. A strong understanding of the plaster modification process will enable better digital pathways for AFO production and can have implications for orthotist training.
